# Automation Inner Speech as an Anthropomorphic Feature Affecting Human Trust: Current Issues and Future Directions

**DOI:** 10.3389/frobt.2021.620026

**Published:** 2021-04-23

**Authors:** Alessandro Geraci, Antonella D'Amico, Arianna Pipitone, Valeria Seidita, Antonio Chella

**Affiliations:** ^1^Robotics Lab, Department of Engineering, University of Palermo, Palermo, Italy; ^2^Department of Psychology, Educational Science and Human Movement, University of Palermo, Palermo, Italy

**Keywords:** inner speech, trust, anthropomorphism, human-automation interaction, human-robot interaction, robot, automation

## Abstract

This paper aims to discuss the possible role of inner speech in influencing trust in human–automation interaction. Inner speech is an everyday covert inner monolog or dialog with oneself, which is essential for human psychological life and functioning as it is linked to self-regulation and self-awareness. Recently, in the field of machine consciousness, computational models using different forms of robot speech have been developed that make it possible to implement inner speech in robots. As is discussed, robot inner speech could be a new feature affecting human trust by increasing robot transparency and anthropomorphism.

## Introduction

In the past years, robots and automation development and implementation have increased exponentially in every context, leading to growing interactions with humans (Merritt and Ilgen, 2008). Robots are now used in different contexts, such as military, security, medical, domestic, and entertainment (Li et al., 2010). Robots, compared with other types of automation (e.g., machines, computers), are designed to be self-governed to some extent to respond to situations that are not prearranged (Lewis et al., 2018). Therefore, the greater the complexity of robots, the higher the importance to focus on factors that influence human–automation interaction (HAI) as their collaboration increases over time (Lee and See, 2004; Schaefer et al., 2016). In this paper, we aim to start the exploration of the role of inner speech in HAI and, in particular, on its role in improving human trust toward automation. For this purpose, we first focus on the concept of inner speech in psychological literature, also examining the first results of its implementation in automation. Then, we discuss the possible role of inner speech as one of the anthropomorphic automation features that may affect human trust in HAI.

## Inner Speech

Inner speech is an everyday covert inner monolog or dialog with oneself, which is essential for human psychological life and functioning because it is linked to reasoning, self-regulation, and self-awareness (Morin, 2012).

For its nature, inner speech is intrinsically dialogic because it involves the verbal perspective of thoughts. It is not an image or a sensation or pure emotion. Non-verbal thinking is not inner speech.

Inner speech takes the form of a monolog if the communication is one-sided, and it takes the form of dialog when it includes more than one perspective. More specifically, the monolog form involves a conversation with oneself, in which only one point of view is expressed, and an answer is not required (Oleś et al., 2020). On the contrary, the dialog form refers to a simulated exchange between two or more “selves” or between oneself and other imaginary interlocutors, in which two or more points of view or perspectives are taken into account (Fernyhough, 2016).

Nowadays, there are many alternative terms used to refer to inner speech, such as inner voice, private speech, inner language, internal dialog, self-talk, covert speech (Loevenbruck et al., 2018). However, the most accepted definition describes inner speech as “the subjective experience of language in the absence of overt and audible articulation” (Alderson-Day and Fernyhough, 2015, p. 931). Interest in inner speech originates from the psychological literature, particularly from the theoretical debate on the relationship between language and thinking and on the role of inner speech for cognitive development.

Watson (1913), the father of behaviorism, equated inner speech with thinking, affirming that external and inner speech share the same structures except for the articulatory components: child's overt speech transitions to covert speech, passing by whispering, simply through a process of reduction of audible volume. Piaget (1959) named inner speech as egocentric speech, emerging during children's playtime, which he believed to be intimately related to action. He considered egocentric speech to have no specific functions and, thus, to be an egocentric thinking expression. In this early stage, the child cannot discern his perspective from others, destined to disappear, giving way to social language gradually.

Vygotsky (1962), on the contrary, attributed great importance to inner speech as one of the most crucial processes for cognitive and social development. According to Vygotsky (1962), inner speech serves multiple cognitive functions, such as problem solving and self-regulation, because it allows using and controlling thought to plan and monitor behaviors and actions.

Vygotsky (1962) also argued that external and inner speech are almost opposites because “external speech is a process of transforming thought into word; it is the materialization and objectification of thought. Inner speech moves in the reverse direction […] it is a process that involves the evaporation of speech in thought” (p. 258). He reasoned that early linguistic, social interaction between the child and the caregivers are gradually internalized and transformed into covert self-directed speech. As internalization progresses, the child becomes more psychologically autonomous and self-regulated because “a function becomes internalized when it can be fulfilled without the immediate collaboration of others” (Larrain and Haye, 2012, p. 6).

Despite Vygotsky's fundamental theoretical contribution to inner speech and its central role in human psychological development, over the years, scientific research has shown little attention to this field (Scott et al., 2013). One reason may be due to a general assumption that inner speech follows overt speech form and structure (McCarthy-Jones and Fernyhough, 2011). Another reason is linked to methodological issues in the assessment methods (Alderson-Day and Fernyhough, 2015) because inner speech can be neither observed directly nor behaviorally (Martin et al., 2018), and it can vary in terms of frequency among people (Ren et al., 2016).

More recently, there has been a renewed interest in inner speech: McCarthy-Jones and Fernyhough (2011), following the Vygotsky perspective, argue that inner speech qualitatively differs from overt speech because it has a dialogic and condensed nature; it engages the presence of other people's voices, and it is involved in self and other evaluations. That is because “talking to oneself can instigate a fictional dialog in which […] people sometimes […] express to a real or imaginary person their reasons for behaving in a given way or for possessing some personal attributes” (Morin, 2004, pp. 212–213). Inner speech can vary in syntax, semantics, and phonology, spanning from a fully expanded speech to a highly condensed form (Fernyhough, 2004).

The interest in inner speech also depends on recognizing its contribution to other cognitive processes, such as working memory (Baddeley and Hitch, 1974). Working memory is one of the most studied crucial cognitive processes because it does not attain the maintenance of information exclusively but also allows the elaboration of incoming information during a complicated task. For instance, reading, reasoning, or taking a conversation are all processes managed by working memory because, at the same time, we have to hold recent information in short-term memory, to recover old information from long-term memory, and to orient our attention toward the incoming information. Inner speech is considered to support the phonological loop, the working memory slave system responsible for the representation of acoustic, verbal, or phonological information. By inner speech (also referred to as rehearsal process), people maintain recent verbal information in memory while new information is being processed or old information is recovered from long-term memory.

Recently, different studies have demonstrated that inner speech is related to various psychological processes: Tullett and Inzlicht (2010) find that suppressing inner speech, using articulatory suppression tasks, increased impulsive responding during go/no-go tasks, concluding that inner speech is involved in self-control abilities. Gade and Paelecke (2019) show inner speech is linked to conflict resolution abilities and cognitive flexibility as they demonstrate that it improved participants' performances in the Simon task. In addition, studies found that expressing overtly one's mental verbalization during a task (i.e., thinking aloud protocol) facilitates problem-solving and reading comprehension because it helps to be more focused and more capable of following a sequence of self-instruction (Short et al., 1991; Kucan and Beck, 1997).

Inner speech is also associated with self-awareness, a multisource psychological ability to orient attention to oneself (Morin, 2011) because “one becomes self-aware when one engages in self-talk (higher-order thought) about one's current mental state and personal characteristics” (p. 212). According to Morin (2012), inner speech allows recognizing different self-facets, representing internal states, and consequently thinking about them.

Psychological studies show that inner speech also has a “dark side” because it is involved in different psychopathological disorders (for reviews, see Alderson-Day and Fernyhough, 2015). It is related to auditory verbal hallucinations, a prominent symptom in psychotic disorders, especially schizophrenia (Waters et al., 2012), which refers to the subjective experience of hearing voices in the absence of a speaking source. The most supported theory states that these symptoms derive from a deficit in self-recognition processes. People fail to recognize their thoughts and behaviors as self-generated, consequently believing them to originate from an external source (Frith and Done, 1988; Bentall, 1990; Waters et al., 2012). Other studies show that inner speech is also involved in anxiety and mood disorders: the process of rumination (i.e., repetitive presence of negative thoughts), which is predominantly verbal, generates a negative emotional and cognitive loop maintaining or intensifying the levels of anxiety and depression (McCarthy-Jones and Fernyhough, 2011; Whitmer and Gotlib, 2012; Alderson-Day and Fernyhough, 2015).

Inner speech has been also studied from a neuroscientific perspective. Some studies show that different brain regions activate when inner speech takes the form of either a monolog or a dialog. In the monolog scenario, the inner speech involves the activation of left frontotemporal regions associated with overt speech production and understanding (e.g., left inferior frontal gyrus, middle temporal gyrus: McGuire et al., 1996; Shergill et al., 2002). On the contrary, the dialogic inner speech involves an extensive neural network between two hemispheres (e.g., left and right superior temporal gyri, posterior cingulate: Alderson-Day et al., 2016).

## Inner Speech as an Emerging Area of Research in AI

Inner speech is also an emerging area of interest in the field of artificial intelligence. Over the last two decades, various computational models have included simulations of different inner speech forms (Reggia, 2013). For instance, Steels (2003) argues that agents' programmed capability of reentering speech production (output) as speech comprehension (input) (i.e., reentrant system) “is useful for detecting and repairing language communication, and thus for pushing language and its underlying meaning toward greater complexity” (p. 11). Similarly, Clowes (2006) argues that inner speech contributes to organizing consciousness. Still, he goes further by also emphasizing the role of inner speech in regulating and shaping ongoing activities and orienting attention. Compared with Steels (2003), Clowes (2006) proposes a self-regulation model in which inner speech could serve “as a scaffold for developing and sustaining cognitive functions beyond the parsing and construction of meaningful and grammatical utterances” (Clowes, 2006, p. 120; see also Clowes, 2007). This model was tested in a series of experiments (Clowes and Morse, 2005) in which groups of agents, implemented with a simple recurrent neural network, had to execute different tasks. The agents in the experimental condition, compared with those in the control condition, were equipped with speech reentrant architectures. Results show that agents with speech reentrance performed better than those who were not programmed with such capability.

Recently, Chella and Pipitone (2020) have proposed a cognitive architecture that uses inner speech to improve robot self-awareness (Chella et al., 2020). It is based on the standard model of mind (Laird et al., 2017) and integrates theoretical contributions on working memory (i.e., phonological loop; Baddeley and Hitch, 1994). The architecture is composed of two main layers: a motor-perception layer and a memory layer. The motor-perception layer enables the robot to perceive information and to take actions: the perception regards data from both itself (e.g., emotions, body, and beliefs and general inner state) and the outside world (the facts in the environment). Along the same line, the motors act on the external entities (e.g., to pick objects, to identify locations) or internal entities (e.g., to self-regulate, to appraise a situation).

The memory layer includes long-term memory, which stores both domain knowledge (declarative memory) and behavioral information (procedural memory), and the working memory system, which models the cognitive functions and processes. The working memory elaborates information from the motor-perception layer by integrating them with information retrieved from the long-term memory.

The architecture of inner speech fits the Baddeley and Hitch's (1994) components into both layers: more specifically, inner speech is modeled as a loop between the phonological store (which briefly holds verbal and written linguistic information) that is a subcomponent of working memory, and the covert articulator (which is responsible to produce linguistic information and then reenter it in the phonological store) located in the motor-perception layer. In Chella and Pipitone's (2020) architecture, inner speech is not just based on a speech reentrance for memorizing data, by which the output word is simply reentered as an input in the phonological store. On the contrary, when the robot processes a word, it is rehearsed by the phonological store, and it is integrated with the information retained in the long-term memory system so that the initial input word is extended in a more elaborated way. All these contributions are aimed at implementing inner speech in automation to improve its functioning (self-awareness, self-regulation, or performance).

No studies, however, have been performed so far with the aim to test if automation equipped with inner speech may affect the quality of HAI and, in particular, human trust toward automation.

## The Role of Trust in HAI

Trust research is a well-established field of scientific knowledge that has collected contributions of different disciplines over the years (e.g., psychology, philosophy, sociology; Paliszkiewicz, 2011) and has focused particularly on the field of HAI. In psychology, trust is a multidimensional concept with no universal definition, which generally refers to an underlying psychological state affected by both cognitive and affective processes (Lewis and Weigert, 1985; McAllister, 1995; Cummings and Bromiley, 1996; Kramer, 1999; Chowdhury, 2005; Johnson and Grayson, 2005; Paliszkiewicz, 2011). Cognitive trust refers to an individual's conscious decision to trust based upon one's beliefs and knowledge about a partner's reliability and competence (McAllister, 1995; Paliszkiewicz, 2011). On the contrary, affective trust stems from interpersonal and emotional bonds, mostly based on the feelings of security, care, and mutual concern (McAllister, 1995; Johnson and Grayson, 2005). From a functional perspective, trust serves as a psychological mechanism for the reduction of social complexity through the formation of expectations and beliefs about others' intentions and behaviors (Luhmann, 1979; Lewis and Weigert, 1985; Rompf, 2014). Lewis and Weigert (1985) state that rational prediction requires time and mental resources for collecting and processing information to determine highly probable outcomes, and thus, trust may be an efficient alternative. Indeed, “by extrapolating past experiences into the future, individuals save the cognitive resources which would be otherwise needed for the search of information and its deliberate processing” (Rompf, 2014, p. 98). Within the psychological literature, trust definitions highlight two key elements: on one side, trust activates positive attitudes, expectations, or confidence in the trustee (Rotter, 1967; Corritore et al., 2003; Lee and See, 2004); on the other, it implies a willingness to put oneself at risk or in a vulnerable state (Mayer et al., 1995; Kramer, 1999; Lee and See, 2004). Muir (1987) states that trust is generally defined as an expectation of or confidence in another and always has a specific referent. Indeed, it involves a relationship between “a trustor A that trusts (judges the trustworthiness of) a trustee B concerning some behavior X in context Y at a time T” (Bauer and Freitag, 2017, p. 2). Moreover, trust is dynamic because it develops and changes over time. Still, it is not a linear process: It may evolve as well as it may deteriorate through a process of loss and repair in response to individual, social, and environmental factors (Paliszkiewicz, 2011; Fulmer and Gelfand, 2013). Similarly, in HAI literature, trust is generally defined as an “attitude that an agent will help achieve an individual's goals in a situation characterized by uncertainty and vulnerability” (Lee and See, 2004, p. 54), and it is considered one of the main factors linked to automation use (Parasuraman and Riley, 1997; Lee and See, 2004; Merritt and Ilgen, 2008; Lewis et al., 2018). Accordingly, trust plays a crucial role in reliance between humans and automation, allowing the latter to take on a full collaborative partner (Lee et al., 2013; Hoff and Bashir, 2015). HAI studies found that people tend to rely on automation they trust and reject those who they do not trust (Muir and Moray, 1996; Lewandowsky et al., 2000; Lee and See, 2004). According to Muir (1987), the same elements that serve as a basis for trust between individuals may affect HAI. However, whereas in human–human interaction, trust is affected by both cognitive and affective processes (Lewis and Weigert, 1985; Mayer et al., 1995), in HAI, trust is considered to be affected predominantly by cognitive aspects because the machine is expected to reach standard performances (Muir, 1994; Merritt and Ilgen, 2008; Lewis et al., 2018).

Lee and Moray (1992) propose that, in HAI, trust is based on three factors: performance, process, and purpose (see also Lee and See, 2004). Performance refers to the automation's capabilities and competencies to achieve the operator's goals. The process focuses on the algorithms and operations that govern the conduct of automation. Purpose concerns the designer's intent behind the automation development. These factors address the user's perception and knowledge of what automation does, how it works, and why it was developed. Merritt and Ilgen (2008) propose a slightly different classification, suggesting four machine characteristics that may affect human trust: competence (i.e., automation's abilities to perform well), responsibility (i.e., automation's functioning information availability to the user), predictability (i.e., automation's behavior consistency), and dependability (i.e., automation's behavior consistency over time). Trust is crucial in HAI because it is related to both misuse and disuse: misuse occurs when humans over-trust automation, relying excessively on its abilities compared with what it can execute, whereas disuse refers to the lack of trust in automation's capabilities so that the human chooses simply not to use it, resulting in a worse outcome (Parasuraman and Riley, 1997; Lee and See, 2004). Both misuse and disuse, especially for high-risk situations, may have catastrophic consequences, such as a plane crash (Lee and See, 2004; Lyons and Stokes, 2012). If trust is appropriately calibrated, which is when human trust correctly matches the true capabilities of the automation (Lee and See, 2004), misuse and disuse may be avoided, enabling an adequate, optimal, and safe HAI (Hoff and Bashir, 2015; Lewis et al., 2018). Nevertheless, there is some evidence that people rely on automation due to a bias that it makes fewer mistakes than humans do, which, in turn, may lead people to reduce reliance on automation as they perceive and remember more automation error and omission than in humans (Dzindolet et al., 2002; Madhavan and Wiegmann, 2004). However, the extent to which an automation error produces changes in the human trust level is still unclear: for instance, trust levels may drop rapidly in response to the first automation errors (Sauer et al., 2016), but they may also decrease when automation fails at humans' easily perceived tasks (i.e., easy error hypothesis; Madhavan et al., 2006) so that the operator infers that, most likely, the automation will not be able to perform a difficult and complex task either. Another similarity in trust among humans and in HAI refers to its development. In this respect, three main phases have been identified: trust formation, dissolution, and restoration (Rousseau et al., 1998; Kim et al., 2009; Fulmer and Gelfand, 2013). Trust formation starts when a trustor chooses to trust a trustee based on the perceived trustworthiness (i.e., ability, benevolence, integrity; Mayer et al., 1995). If trust is repeatedly violated, the trustor decreases trust levels in the trustee, entering the dissolution phase. The restoration phase happens when the trustor deliberately adopts repair strategies that allow trust levels in the trustee to increase again, eventually stabilizing. These three phases are not necessarily linear and straightforward because trust, at some point in time, may be the result of ongoing violations and repairs.

All these studies highlight that most of the psychological processes involved in human–human interaction can also be accounted for in HAI even though automation may be affected by certain biases because, for instance, they are expected to be infallible. Nevertheless, knowledge of these processes is crucial for improving human trust calibration toward the automation so that their expectancies reflect the actual characteristics and capabilities of the automation, eventually enhancing human–automation collaboration.

## May Automation Inner Speech Affect Human Trust?

In the HAI literature, most researchers agree that trust dynamically emerges from the exchange of the distinct features of the operator, the machine, and the specific environment in which the interaction takes place (Hancock et al., 2011; Hoff and Bashir, 2015; Schaefer et al., 2016; Kessler et al., 2017; Lewis et al., 2018). Two extensive meta-analyses carried out by Hancock et al. (2011) and Schaefer et al. (2016) identify three main components affecting trust in human–robot interaction and in HAI: human-related factors, automation/robot–related factors, and environment-related factors (see Table 1).

**Table 1:** Comparison of human–automation and human–robot trust models.

**Human–Automation trust factors(Schaefer et al., 2016)**	**Human–Robot trust factors (Hancock et al., 2011)**
**I–Human-Related**	**I–Human-Related**
Cognitive factors (e.g., knowledge and ability)	Ability-Based (e.g., expertise and competency)
Emotive factors (e.g., comfort and confidence)	Characteristics (e.g., personality, age, and gender)
Traits (e.g., personality, age, and gender)	
States (e.g., stress and fatigue)	
**II–Automation-Related**	**II–Robot-Related**
Features (e.g., intelligence and anthropomorphism)	Attribute-Based (e.g., personality and robot type)
Capability (e.g., behavior, error, and feedback)	Performance-Based (e.g., transparency and failures)
**III–Environment-Related**	**III–Environment-Related**
Team collaboration (e.g., membership and culture)	Team collaboration (e.g., membership and culture)
Task/Context (e.g., task type and uncertainty)	Tasking (e.g., task type and task complexity)

In both models, human-related factors take into account individual differences (e.g., personality traits, age, and gender), emotions (e.g., comfort, confidence), and cognition (e.g., expertise, expectancy, and abilities); automation/robot–related factors consist of characteristics such as personality and anthropomorphism and abilities such as behavior, failures, and errors; environment-related factors include elements such as culture, group membership, context, and task.

Our idea is that inner speech could be one of the automation-related factors influencing trust. Inner speech, in particular, could influence the anthropomorphism of automation.

Anthropomorphism is “the act of attributing humanlike qualities to non-human organisms or objects” (DiSalvo and Gemperle, 2003, p. 68), and it incorporates a wide range of human characteristics from poor appearance and basic behaviors to real-like aesthetics and social communication (Pak et al., 2012). In the past years, researchers argue that people might respond socially to computers and other technologies using the same social rules applied to human interaction (Nass et al., 1995; Reeves and Nass, 1996). This phenomenon, named ethopoeia, is defined as an “assignment of human attitudes, intentions, or motives to non-human entities” (Nass et al., 1993, p. 111). Indeed, people might anthropomorphize those robots that behave typically like humans during social interaction (e.g., stare, gestures, etc.; Duffy, 2003). In an experimental study, Salem et al. (2013) confirmed that non-verbal behaviors during social communication affected anthropomorphic inferences about a robot. They found that the robot's coverbal hand and arm gestures during interaction increased participants' anthropomorphic perceptions and likeability, and this effect was greater for the incongruent condition in which the robot's speech and gesture partially matched. Short et al. (2010) find that even the display of a cheating behavior by the robot during a rock–paper–scissors game increased participants' social engagement with the robot and the attributions of mental states.

Robots' appearance and design also have an important influence on their perceived human-likeness: the presence of certain features (i.e., nose, eyelids, and mouth) and width of the head compared with height increases the levels of robot anthropomorphism (DiSalvo et al., 2002). Similarly, Hinds et al. (2004) show that participants delegated responsibility and relied more on the human-like robot coworker (e.g., face, nose, eyes, mouth, and hair) compared with the machine-like robot when performing a task. Several studies show that increasing the anthropomorphism of an interface enhances people's trust in automation aids even when the information presentation and reliability are identical for other non-anthropomorphic interfaces (de Visser et al., 2012; Pak et al., 2012). In addition, van Pinxteren et al. (2019) find that social service robot anthropomorphism (i.e., gaze turn-taking cues) account for significant variation of trust scores. In addition to how an agent looks, people may also respond to how it sounds. Indeed, people tend to trust more those systems that produce human speech rather than synthetic speech (Stedmon et al., 2007; Eyssel et al., 2012).

Similarly, we suggest that automation equipped with an overt mental verbalization system, which reproduces human inner speech, could make it easier for people to attribute human-like qualities to automation, ultimately enhancing human trust in robots.

In our opinion, this might happen for different reasons, which depend both on the effects of monologic/dialogic inner speech in automation's behavior and the overt or covert nature of inner speech.

Considering the first point, we already discussed that some scholars in the field of AI started to implement inner speech in automation to improve their performance, self-regulation, self-awareness, and consciousness (Steels, 2003; Clowes, 2007; Reggia, 2013; Chella et al., 2020).

Thus, a robot equipped with monologic/dialogic inner speech is expected to be more performative, more self-regulated, more aware of its behaviors, and, finally, more similar to humans.

If it is true, as described in the psychological literature, that monologic inner speech influences thinking and reasoning (Vygotsky, 1962; Morin, 2012; Alderson-Day and Fernyhough, 2015; Gade and Paelecke, 2019), automation implemented with inner speech should improve its performance in making decisions that are more complex. For instance, using inner monolog, the automation could self-guide itself in preparing purposes, goals, plans, and test them before acting as people do when self-guiding themselves in tasks that require attention.

Self-guidance and self-instruction could allow automation, similarly to humans, to become more aware of its choices and actions and more self-regulated.

The dialogic inner speech could also influence the robot performance and behavior: if it is true that it helps people to reframe their opinions, taking the others' perspective, and to solve problems, considering them from different points of view, automation equipped with dialogic inner speech should be more able in both perspective taking and problem solving. Besides this, humans could improve their trust in this type of automation.

Considering the second point, we suggest that also the overt or covert nature of inner speech could influence trust. Indeed, covert inner speech is a phenomenon that sometimes is automatic and not visible to others (Martin et al., 2018).

As already described, the process of transforming thinking in voice facilitates problem solving and reading comprehension (Short et al., 1991; Kucan and Beck, 1997). Thus, automation equipped with overt inner speech may perform better than automation equipped with covert inner speech. It could happen because overt inner speech may be reprocessed by automation's vocal recognition systems, sending to the central processor two different inputs: one is the plan of action sent to the output language system, and the second is the plan of action reprocessed by the language input system. These two different inputs may help the automation to have more control and awareness of the sequence of activities planned or executed and, finally, to perform better. Again, the better the automation performance, the higher the human trust.

We also suggest that overt inner speech may help to improve HAI and, in particular, human–automation trust. Indeed, cognitive processes of automation equipped with robot inner speech would be more transparent and more understandable to humans.

For example, during the execution of a cooperative task between the automation and the human, overt inner speech would manifest automation's “mental” processes (e.g., reasoning that underlies its actions, motivation, goals, and plan of actions). In this way, automation becomes a transparent and overt system, letting humans better understand how it works and what determines its behavior.

Mind perception is how people discern between human and nonhuman agents and consists of two core dimensions: (1) agency, e.g., self-control, memory, planning, and communication, and (2) experience, e.g., pain, pleasure, desire, joy, consciousness (Gray et al., 2007). Consequently, transparency in automation cognitive functioning may help people to increase human-like attributions. A recent study shows that transparency about the robot's lack of human psychological processes and abilities reduced children's anthropomorphic tendencies and trust (van Straten et al., 2020). Therefore, transparency may improve automation anthropomorphism, and as described before, automation anthropomorphism influences trust.

Moreover, inner speech makes cooperation more robust because the robot could evaluate different strategies for going out from a stalemate. For example, suppose, for some reason, a step of the whole task to execute is not feasible (e.g., an object to pick is placed in an unattainable position). In that case, the self-dialog may enable the robot to reflect on possible alternatives for reaching the same goal. Meanwhile, the robot can involve the partner in the new planning, and further turns of interaction enrich the cooperation. By hearing the inner reasoning and the evaluation of the robot, the partner may gain more confidence. The robot's behavior becomes not unpredictable, thus affecting the growth of trust.

Overt inner speech could also have a role in the process of trust development. Indeed, humans would probably be more facilitated in developing trust in an overt system, so when dissolution occurs due to errors by automation, a human may better understand why the errors occurred; this, in turn, could facilitate the trust restoration. In this regard, Kim and Hinds (2006) show that, when a robot shows high transparency during an assembling task by explaining its unexpected behaviors, people tend to blame it less compared with those robots who have less transparency.

## Conclusion

In this paper, we propose the new idea that inner speech could be one of the functions to be implemented in automation to improve its levels of reasoning, thinking, self-control, self-awareness, and, finally, performance. Moreover, we propose that overt inner speech, allowing people that interact with automation to know and understand the reasoning processes that underlie its behaviors, might influence the level of transparency of automation and, finally, its level of anthropomorphism.

Both performance and anthropomorphism are two essential factors influencing human–automation trust, and for these reasons, we consider the implementation of inner speech as a new important frontier for increasing the quality of HAI and the trustworthiness of automation. On the other hand, some promising cognitive architectures for implementing inner speech have already been proposed. Still, no studies have been performed so far for testing to what extent automation using inner speech affects human trust.

In the end, this paper's discussion is speculative, and it is based exclusively on theoretical considerations based on empirical evidence from different research fields. However, we believe that future studies on inner speech may represent a new frontier in robotics and AI, and in this sense, we hope that our idea may stimulate further research study in this area.

## Author Contributions

All authors listed have made a substantial, direct and intellectual contribution to the work, and approved it for publication.

## Conflict of Interest

The authors declare that the research was conducted in the absence of any commercial or financial relationships that could be construed as a potential conflict of interest.
